# Application of Minimally Invasive Oral Swab Samples for qPCR-Based Sexing in Neognathae Birds

**DOI:** 10.3390/vetsci12010073

**Published:** 2025-01-20

**Authors:** Maria-Carmen Turcu, Anamaria Ioana Paștiu, Lucia-Victoria Bel, Anca-Alexandra Doboși, Dana Liana Pusta

**Affiliations:** 1 Department of Genetics and Hereditary Diseases, Faculty of Veterinary Medicine, University of Agricultural Sciences and Veterinary Medicine Cluj-Napoca, 400372 Cluj-Napoca, Romania; maria-carmen.turcu@usamvcluj.ro (M.-C.T.); anca.dobosi@student.usamvcluj.ro (A.-A.D.); dana.pusta@usamvcluj.ro (D.L.P.); 2 New Companion Animals Veterinary Clinic, Faculty of Veterinary Medicine, University of Agricultural Sciences and Veterinary Medicine Cluj-Napoca, 400372 Cluj-Napoca, Romania; lucia.bel@usamvcluj.ro

**Keywords:** birds, qPCR, molecular sexing, *CHRNA6*, *DDX4*, *VPS13A*, *LPAR1*, and *TMEM161B* genes

## Abstract

Birds are inherently social creatures that rely on pairing to enhance their well-being. Since many bird species lack obvious physical differences between females and males, sex identification is essential for ensuring their welfare. Additionally, early determination of the sexes of birds is crucial for their breeders, especially considering that most companion birds do not display clear sexual characteristics. Molecular genetic sexing has been demonstrated to be the most reliable method for determining the sexes of monomorphic birds. The objective of the present study was to demonstrate rapid, effective, and precise identification of sex in birds through quantitative real-time PCR (qPCR) using samples obtained via a minimally invasive technique (oral swabs). This qPCR method assesses variations in gene copy numbers within conserved Z-specific genes such as *CHRNA6*, *DDX4*, *VPS13A*, *LPAR1*, and *TMEM161B*, which are absent from the W chromosome. A total of 34 samples were included in this study from the following 17 bird species: domestic pigeon (*Columba livia domestica*), domestic chicken (*Gallus gallus domesticus*), domestic goose (*Anser anser f domesticus*), domestic duck (*Anas platyrhynchos domesticus*), Mute swan (*Cygnus olor*), Budgerigar (*Melopsittacus undulatus*), Lovebird (*Agapornis roseicollis*), Cockatiel (*Nymphicus hollandicus*), Red-rumped parrot (*Psephotus haematonotus*), Rose-ringed parakeet (*Psittacula krameri*), African grey parrot (*Psittacus erithacus*), domestic Canary (*Serinus canaria forma domestica*), Goldfinch (*Carduelis carduelis major*), Gouldian Finch (*Chloebia gouldiae*), Red Siskin (*Carduelis cucullata*), Australian Zebra Finch (*Taeniopygia castanotis*), and Common buzzard (*Buteo buteo*). The results proved *that* the *CHRNA6*, *DDX4*, *VPS13A*, *LPAR1*, and *TMEM161B* genes can reveal the sexes in the *Neognath* birds tested.

## Simple Summary

Birds are social creatures, and identifying their sexes is essential for their breeders to provide for their welfare and for breeding, especially since many species lack visible sexual dimorphism. Molecular genetic sexing using real-time PCR (qPCR) is one of the most reliable methods for sex determination in monomorphic birds. This study aimed to demonstrate the effectiveness of qPCR in sexing *Neognathae* birds using minimally invasive oral swab samples. By analyzing five conserved Z-specific genes (*CHRNA6*, *DDX4*, *VPS13A*, *LPAR1*, and *TMEM161B*), this study successfully sexed paired male and female samples obtained from 17 bird species across six orders. At least one of these genes was effective in determining sex in all tested species. These findings establish qPCR using oral swab samples as a practical and less invasive method for avian sex determination, with potential applications in molecular biology and conservation. Future studies should evaluate its use across additional species and refine the technique for improved accuracy and ease of use.

## 1. Introduction

Modern birds originated around 66 million years ago and have since diversified into more than 10,000 species worldwide. Despite their diverse appearances, many birds exhibit minimal sexual dimorphism, especially in juveniles but also in some adults [1]. Social bonding is crucial for birds’ welfare, but accurate sex identification remains challenging in captive species where physical differences between males and females are subtle or absent. Birds are ranked as the fourth most popular pet in the US [2,3] and the third most popular in the EU [4,5]. Captive-bred birds exhibit behavior and physiology closely resembling their wild counterparts [6], highlighting the minimal variation across generations in captivity.

Genetic sexing of birds plays a crucial role in fields such as behavioral medicine, conservation, wildlife bird management, captive breeding programs, and evolutionary research [7,8]. Traditional methods of sexing birds, such as observing secondary sexual characteristics, are often unreliable and established late due to the birds’ physiology, and endoscopic sexing, though quite accurate, can only be performed later in life after sexual maturity, is invasive, and requires anesthesia. In contrast, molecular sexing stands out as the most accurate and non-invasive method, and it allows for determination of sex early in a bird’s life [9]. Molecular sexing in birds focuses on identifying sexual chromosome characteristics, offering a non-invasive alternative to classical methods like celioscopy (endoscopy). This method is considered the safest, as it poses no risk of harm or infection to the birds during sample collection [8,9,10,11].

Molecular sex determination in birds is chromosomal, where females are heterogametic (ZW) with distinct Z and W chromosomes, while males are homogametic (ZZ) with two Z chromosomes. In *Neognathae* birds, sex chromosomes typically exhibit heteromorphism characterized by smaller, mostly heterochromatic W chromosomes [12]. However, significant variation exists in the size and distribution of heterochromatin among W chromosomes [13]. Recent studies have unveiled diverse patterns in avian sexual chromosomes, including autosome–sex chromosome fusions observed in several passerines [14] and multiple sex chromosomes in penguins [15].

Functional genes on the W chromosome have been lost due to limited recombination between sex chromosomes Z and W, but only in the pseudoautosomal regions. As a result, there are significant differences between bird species in the degrees of Z-W differentiation, W chromosome degradation, and Z-W recombination [16,17]. The chromo-helicase-DNA binding protein (CHD1) gene, which is conserved across avian species, facilitates sex identification using PCR markers that amplify the CHD1-Z and CHD1-W genes’ homologous regions. Differences in intron length distinguish these genes: males typically show copies of a single gene (with identical lengths due to the two Z chromosomes), while females exhibit two copies (differing in length due to the Z and W chromosomes) [8].

The development of molecular methods for bird sex identification was propelled by the discovery of distinct CHD1 gene gametologues on the Z and W chromosomes characterized by differences in intron size and the absence of autosomal copies or pseudogenes [10,18]. Initially, PCR-based assays targeting these gametologues revolutionized sexing by generating distinct banding patterns: females exhibit two bands, while males display a single band [10,18]. Modifications and lineage-specific primers have since refined this method [19].

Additionally, alternative PCR-based approaches have emerged, including allele-specific polymerase chain reaction (PCR), restriction fragment length polymorphism (RFLP), single-strand conformation polymorphism (SSCP), capillary electrophoresis, loop-mediated isothermal amplification (LAMP), and real-time PCR with TaqMan probes or high-resolution melting analysis [8,20,21,22]. These advancements emphasize the continuous progress and implementation of accurate molecular techniques for sex determination in birds.

In pursuit of a universal method for avian sex identification, numerous PCR markers targeting genes such as CHD1 [10,18,19], the ATP synthase α-subunit (ATP5A1) [23], the W-linked altered form of protein kinase C-interacting protein (Wpkci) [24], the Nipped-B homolog (NIPBL) [25], Spindlin (SPIN) [26], and RAS p21 protein activator 1 (RASA1) [27] were developed. These genes help distinguish between the homologous regions of sex chromosomes Z and W by detecting length polymorphisms in the introns within these regions [28,29].

Despite its widespread application, molecular sexing by identifying the CHD1 gene using conventional PCR faces challenges in certain avian species. Issues include the preferential amplification of either the CHD1-W or CHD1-Z allele in females, potentially causing misidentification as males when only a single band appears on electrophoretic gels [30]. Standard CHD1 primers may yield inadequate amplification in certain avian species [22,27], and, moreover, variations in banding patterns on electrophoretic gels further complicate molecular sexing techniques [31]. Variations in the size of the CHD1-Z allele can also result in incorrect identification as females [32,33], and subtle differences in size between the two gametologues can complicate accurate sex identification when they are beyond the detection limit of electrophoresis [34]. Additionally, optimizing PCR conditions such as the annealing temperature and primer selection for each species can be a time- and resource-consuming process [35]. These obstacles and challenges led to adaptations in molecular sexing techniques to ensure accurate results across diverse avian species. The limitations identified in conventional PCR methods have spurred the refinement and advancement of molecular sexing approaches, notably quantitative real-time PCR (qPCR). This innovative technique aims to achieve improved precision and reliability in sex determination in birds [8,36].

Quantitative real-time PCR (qPCR) is a targeted method for sexing birds. This approach involves real-time amplification and quantification of DNA using fluorescent probes or dyes, which monitor the PCR process in real time [8]. Non-invasive and minimally invasive sample collection methods, such as buccal swabs and feather collection, are preferred over blood collection for PCR in avian studies, reducing stress and potential harm to birds. It is particularly accurate for monomorphic species, including young or juvenile birds [37,38,39]. Beyond aviculture, conservation biology, and veterinary practice, qPCR supports precise breeding management by ensuring accurate bird pairing [8]. Compared to traditional methods, qPCR provides faster, more reliable results with quantitative data while minimizing sampling risks, solidifying its role as a cornerstone of modern avian sex determination methodologies [17,40].

The main aim of this study was to demonstrate rapid, efficient, and accurate determination of bird sex using qPCR with oral swab samples collected through minimally invasive methods. Additionally, this research aimed to evaluate the effectiveness of qPCR-based sex identification in *Neognathae* birds, including parrots, canaries, finches, domestic pigeons, land fowl, waterfowl, and buzzards, by targeting conserved Z-specific genes such as *CHRNA6*, *DDX4*, *VPS13A*, *LPAR1*, and *TMEM161B*, which are absent from the W chromosome. This study aimed to validate the efficiency of these genes by analyzing paired male and female samples from 17 bird species across six orders (*Accipitriformes*, *Galliformes*, *Anseriformes*, *Columbiformes*, *Passeriformes*, and *Psittaciformes*), confirming their reliability for sex determination in the tested *Neognathae* species.

## 2. Materials and Methods

### 2.1. Sample Collection

From 2022 to 2023, a total of 17 paired samples (males and females) were collected from the following 17 bird species: Common buzzard (*Buteo buteo*), domestic chicken (*Gallus gallus domesticus*), Mute swan (*Cygnus olor*), domestic goose (*Anser anser f domesticus*), domestic duck (*Anas platyrhynchos domesticus*), domestic Canary (*Serinus canaria forma domestica*), Australian Zebra Finch (*Taeniopygia castanotis*), Gouldian Finch (*Chloebia gouldiae*), Goldfinch (*Carduelis carduelis major*), Red Siskin (*Carduelis cucullata*), domestic pigeon (*Columba livia domestica*), African grey parrot (*Psittacus erithacus*), Rose-ringed parakeet (*Psittacula krameri*), Cockatiel (*Nymphicus hollandicus)*, Red-rumped parrot (*Psephotus haematonotus)*, Lovebird (*Agapornis roseicollis*), and Budgerigar (*Melopsittacus undulatus*) (Table 1). A minimum of two swabs per bird were sampled using sterile cotton swabs (Prima, Taizhou Honod Medical Co., Ltd., Linhai, China) following the protocol outlined by Handel et al. [41]. The oral swab samples were collected during routine examinations of live birds (*Anseriformes*, *Galliformes*, *Psittaciformes*, and *Passeriformes*) or from cadavers (*Columbiformes* and *Accipitriformes*) at the New Companion Animals veterinary clinic of the Faculty of Veterinary Medicine, University of Agricultural Sciences and Veterinary Medicine of Cluj-Napoca, Romania. Written consent was obtained from the owners for all procedures. The samples were handled with surgical gloves, labeled, and stored at −20 °C until processing. All individual samples included in the present study were previously tested for sex identification using the conventional PCR-based methods described by Griffith et al. [9] and Ito et al. [20].

### 2.2. DNA Extraction and qPCR

DNA was extracted from the 34 oral swab samples collected from the birds using a DNeasy Blood & Tissue Kit (Qiagen, Hilden, Germany) following the manufacturer’s tissue protocol. For DNA extraction, the protocol recommended for tissue samples was used. The swabs were transferred to 1.5 mL Eppendorf tubes with sterile scissors. DNA concentrations were quantified using an ND-1000 spectrophotometer (NanoDrop Technologies, Wilmington, DE, USA).

Real-time PCR was used to determine the variation in the Z-specific gene copy number between males (ZZ) and females (ZW), as previously reported by Mazzoleni et al. [40] and Rovatsos et al. [42]. Z-specific genes (*CHRNA6*, *DDX4*, *VPS13A*, *LPAR1*, and *TMEM161B*) and autosomal genes (*MECOM*, *GGPS1*, and *KIAA1429*) were used. Briefly, all DNA samples were amplified in triplicate on an Azure Cielo™ Real-time PCR system (Azure Biosystems, Dublin, CA, USA). Real-time PCR amplification was carried out in a 15 μL reaction mixture consisting of 7.5 μL of Go Taq DNA Polymerase (Promega, Madison, WI, USA), 0.2 μL of CXR (Promega, USA), 15 pM of each primer [40], and 2 ng of a DNA template. The cycling conditions were as follows: initial denaturation for 3 min at 95 °C, followed by 15 s at 95 °C, 30 s at 56 °C, and 30 s at 72 °C (44 cycles). The melting curve program began with initial denaturation for 15 s at 94 °C, followed by cooling to 65 °C. Then, fluorescent measurements were taken every 0.1 °C from 65 °C to 95 °C. The crossing point (Cp) values were calculated using Azure Cielo Manager software (version 1.0.8.12).

Each target gene’s dosage was calculated using the Cp values and was then normalized in accordance with the dosage of the autosomal reference gene *MECOM* from the same DNA sample. The calculation formulas were previously described by Mazzoleni et al. [40] and Rovatsos et al. [42]. R (the target-to-reference gene dose ratio) = 2^CpMECOM^/2^Cp gene^, and r (the relative gene dose ratio between the sexes for each gene) = R_female_/R_male_. For autosomal genes, r should be around 1.0, and for Z-linked genes r should be around 0.5.

## 3. Results

**Figure 1:**
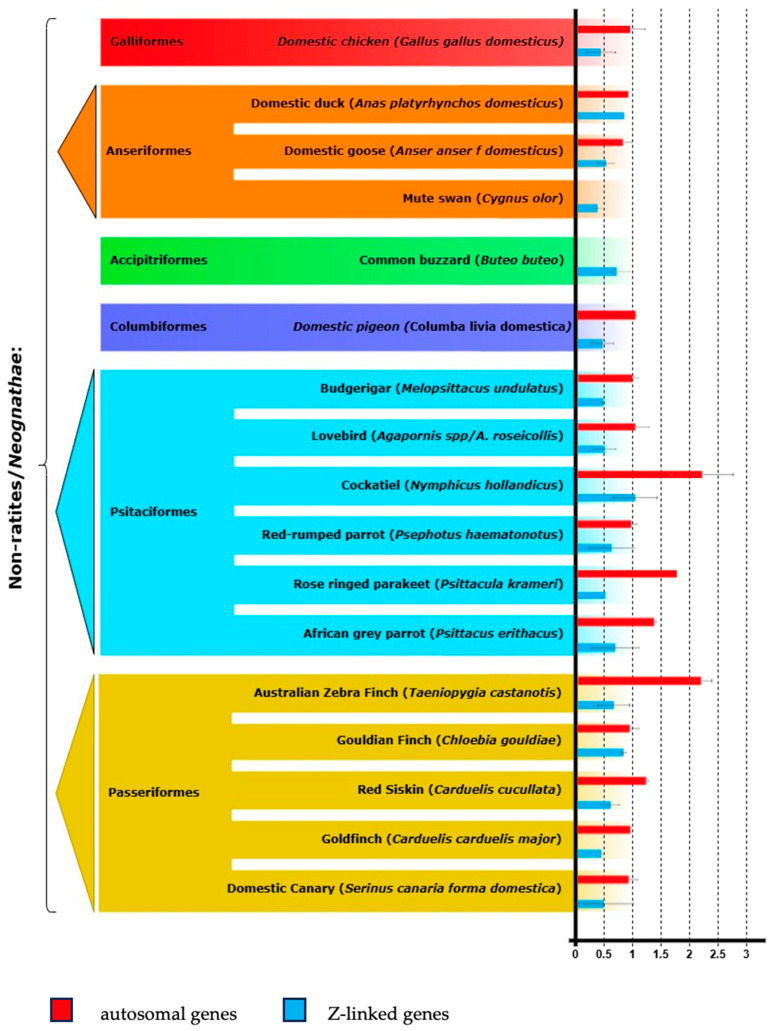
The average relative gene dose ratios between males and females for Z-specific genes (blue) (*CHRNA6*, *DDX4*, *VPS13A*, *LPAR1*, and *TMEM161B*) and autosomal genes (red) (*GGPS1* and *KI-AA1429*) are shown. The *MECOM* gene was used to normalize the real-time PCR values. (Figure adapted from [40]).

The DNA concentrations obtained from the oral swab samples ranged from 5.8 ng/μL to 47.6 ng/μL, with a 260/280 ratio exceeding 1.8. At least one pair of primers specific to Z-linked genes was successfully amplified in each of the species tested (Figure 1 and Table S1). The Z-linked genes that were successfully amplified in most of the tested species were *TMEM161B* and *DDX4* (14/17 species), followed by *CHRNA6* (13/17), *LPAR1* (12/17), and *VPS13A* (7/17) (Table S1). In the domestic ducks and Gouldian finches, we obtained approximatively equal copy numbers between the sexes.

## 4. Discussion

In this study, the genes *CHRNA6*, *DDX4*, *VPS13A*, *LPAR1*, and *TMEM161B* were demonstrated to be effective for qPCR sex determination in birds across six orders: *Accipitriformes*, *Galliformes*, *Anseriformes*, *Columbiformes*, *Passeriformes*, and *Psittaciformes*. We successfully sexed paired male and female samples from 17 species of *Neognathae* birds. Among the five Z-linked genes tested, *TMEM161B*, *DDX4*, and *CHRNA6* showed efficient amplification in almost all the bird species tested. However, *VPS13A* and *LPAR1* were shown to be inefficient in domestic duck and Common buzzard.

It is important to mention that not all Z-linked genes amplify successfully across all bird species [40]. A larger study involving 70 bird species from 19 orders identified *CHRNA6*, *DDX4*, and *TMEM161B* as reliable markers for sex determination in *Neognathae* birds, consistent with our findings. However, that study also identified *LPAR1* and *VPS13A* as effective markers, which contrasted with our results, where these genes showed inefficiencies across the tested species. This difference highlights the variability in Z-linked gene amplification between studies and underscores the importance of validating markers across diverse species.

Expanding on the methods outlined by Mazzoleni et al. [40] and Rotvasos [42], Petrou et al. [17] proposed an innovative approach for sex determination in birds, which also relies on quantifying the numbers of gene copies in conserved Z-linked and autosomal genes. This method uses the quantitative values obtained from qPCR to construct a logistic regression model for sex identification. The main benefit of this approach lies in its ability to overcome limitations associated with pipetting errors and the inherent biological variations among different bird species, offering a more reliable and precise sexing method [17].

The success rate of genetic amplification by qPCR is often influenced by the quality of the extracted DNA [43]. In the present study, minimally invasive oral swabs were used as the DNA source, demonstrating high efficiency when tested by the qPCR methodology. Previous studies have shown the effectiveness of oral swabs as a minimally invasive sampling method for conventional PCR techniques [37,38,39]. The bird species analyzed in this study were previously sexed successfully by the authors using conventional PCR with samples from oral swabs, feathers, and blood, with superior results obtained from the blood and oral swabs. However, the application of qPCR in this research demonstrated greater precision and amplification success compared to conventional PCR, highlighting its advantages as a reliable and robust method for molecular sexing in birds.

Our study had one main limitation: the variation in the DNA concentration and purity among the samples. These factors can affect the reliability of qPCR amplification. However, despite these challenges and the fact that the DNA samples were not selectively chosen for ideal purity, the results were promising. At least one of the five tested Z-linked genes was successfully amplified in each of the samples, enabling reliable qPCR-based sex identification. These findings emphasize the robustness of this method, even in the presence of potential obstacles related to DNA quality. The successful amplification of these genes, despite the variations in DNA quality, demonstrated that this technique is effective for accurate sex determination across the bird species tested. This highlights the potential to use qPCR-based approaches to sex birds, even when confronted with inherent biological variability in samples.

While significant progress has been made in the field of avian sex determination, no single marker or method has been proven to be universally effective for sexing all bird species. Quantitative real-time PCR (qPCR) has emerged as a highly accurate and reliable technique, particularly when utilizing multiple markers or genes [17,28,29]. This approach ensures greater efficacy in molecular sexing by accounting for the genetic diversity across avian species. As research continues to evolve, the refinement and combination of these techniques hold promise for achieving more universally applicable methods for avian sex determination.

## 5. Conclusions

This study demonstrated the applicability of qPCR for sexing *Neognathae* birds. Furthermore, our findings established that oral swab samples are a viable option for molecular sex identification through qPCR. This approach offers a practical and less invasive alternative for avian sex determination, contributing to the broader field of avian molecular biology and conservation efforts. Using the qPCR method detailed by Mazzoleni et al. [24], which relies on conserved Z-specific genes, we successfully sexed paired male and female samples from 17 bird species across six orders: *Accipitriformes*, *Galliformes*, *Anseriformes*, *Columbiformes*, *Passeriformes*, and *Psittaciformes*. At least one of the *CHRNA6*, *DDX4*, *VPS13A*, *LPAR1*, or *TMEM161B* genes was effective for sex determination in these *Neognathae* birds. Future research should explore the broader applicability of this method across different bird species and refine the technique for even greater accuracy and ease of use.

## Figures and Tables

**Table 1:** Numbers of samples from birds in this study.

Order	Species	Male	Female
*Accipitriformes*	*Buteo buteo*	1	1
*Galliformes*	*Gallus gallus domesticus*	1	1
*Anseriformes*	*Cygnus cygnus*	1	1
	*Anser anser f domesticus*	1	1
	*Anas platyrhynchos domesticus*	1	1
*Passeriformes*	*Serinus canaria forma domestica*	1	1
	*Taeniopygia castanotis*	1	1
	*Chloebia gouldiae*	1	1
	*Carduelis cucullata*	1	1
	*Carduelis carduelis major*	1	1
*Columbiformes*	*Columba livia domestica*	1	1
*Psittaciformes*	*Psittacus erithacus*	1	1
	*Psittacula krameri*	1	1
	*Psephotus haematonotus*	1	1
	*Nymphicus hollandicus*	1	1
	*Agapornis fischeri*	1	1
	*Melopsittacus undulatus*	1	1
TOTAL	17	17	17

## Data Availability

All the results of the study are presented in the Manuscript and in the Supplementary Material.

## Supplementary Materials

The following supporting information can be downloaded at https://www.mdpi.com/article/10.3390/vetsci12010073/s1, Table S1: Relative gene dose ratios (r) between females and males for each primer pair and bird species.

## References

[1] Brusatte S.L., O’Connor J.K., Jarvis E.D. (2015). The origin and diversification of Birds. Curr. Biol..

[2] Kidd A.H., Kidd R.M. (1998). Problems and benefits of bird ownership. Psychol. Rep..

[3] Meyers N.M. (1998). Perspectives on pet bird welfare from the pet industry. J. Am. Vet. Med. Assoc..

[4] Davis C. (1998). Appreciating avian intelligence: The importance of a proper domestic environment. J. Am. Vet. Med. Assoc..

[5] Graham D.L. (1998). Pet birds: Historical and modern perspectives on the keeper and the kept. J. Am. Vet. Med. Assoc..

[6] Wyndham E. (1980). Diurnal cycle, behaviour and social organization of the budgerigar *Melopsittacus undulatus*. EMU—Austral Ornithol..

[7] Peng S., Broom D.M. (2021). The sustainability of keeping birds as pets: Should any be kept?. Animals.

[8] Morinha F., Cabral J.A., Bastos E. (2012). Molecular sexing of birds: A comparative review of polymerase chain reaction (PCR)-based methods. Theriogenology.

[9] Morinha F., Travassos P., Seixas F., Santos N., Sargo R., Sousa L., Bastos E. (2013). High-resolution melting analysis for bird sexing: A successful approach to molecular sex identification using different biological samples. Mol. Ecol. Resour..

[10] Griffiths R., Double M.C., Orr K., Dawson R.J. (1998). A DNA test to sex most birds. Mol. Ecol..

[11] Griffiths R. (2000). Sex identification in birds. Seminars in Avian and Exotic Pet Medicine.

[12] Nanda I., Schlegelmilch K., Haaf T., Schartl M., Schmid M. (2008). Synteny conservation of the Z chromosome in 14 avian species (11 families) supports a role for Z dosage in avian sex determination. Cytogenet. Genome Res..

[13] Rutkowska J., Lagisz M., Nakagawa S. (2012). The long and the short of avian W chromosomes: No evidence for gradual W shortening. Biol. Lett..

[14] Sigeman H., Ponnikas S., Hansson B. (2020). Whole-genome analysis across 10 songbird families within Sylvioidea reveals a novel autosome–sex chromosome fusion. Biol. Lett..

[15] Gunski R.J., Cañedo A.D., Garnero A.D.V., Ledesma M.A., Coria N., Montalti D., Degrandi T.M. (2017). Multiple sex chromosome system in penguins (*Pygoscelis*, *Spheniscidae*). Comp. Cytogenet..

[16] Zhou Q., Zhang J., Bachtrog D., An N., Huang Q., Jarvis E.D., Zhang G. (2014). Complex evolutionary trajectories of sex chromosomes across bird taxa. Science.

[17] Petrou E.L., Scott L.C., McKeeman C.M., Ramey A.M. (2024). Molecular sexing of birds using quantitative PCR (qPCR) of sex-linked genes and logistic regression models. Mol. Ecol. Resour..

[18] Fridolfsson A.K., Ellegren H. (1999). A simple and universal method for molecular sexing of non-ratite birds. J. Avian Biol..

[19] Lee J.C., Tsai L.C., Hwa P.Y., Chan C.L., Huang A., Chin S.C., Wang L.C., Lin J.T., Linacre A., Hsieh H.M. (2010). A novel strategy for avian species and gender identification using the CHD gene. Mol. Cell Probes.

[20] Ito H., Sudo-Yamaji A., Abe M., Murase T., Tsubota T. (2003). Sex identification by alternative polymerase chain reaction methods in Falconiformes. Zool. Sci..

[21] Bermudez-Humaran L.G., Chávez-Zamarripa P., Guzmán-Velasco A., Leal-Garza C.H., Montes de Oca-Luna R. (2002). Loss of restriction site DdeI, used for avian molecular sexing, in *Oreophasis derbianus*. Reprod. Domest. Anim..

[22] Chang H.W., Cheng C.A., Gu D.L., Chang C.C., Su S.H., Wen C.H., Cheng C.C. (2008). High-throughput avian molecular sexing by SYBR green-based real-time PCR combined with melting curve analysis. BMC Biotechnol..

[23] Fridolfsson A.K., Cheng H., Copeland N.G., Jenkins N.A., Liu H.C., Raudsepp T., Woodage T., Chowdhary B., Halverson J., Ellegren H. (1998). Evolution of the avian sex chromosomes from an ances- tral pair of autosomes. Proc. Natl. Acad. Sci. USA.

[24] O’Neill M., Binder M., Smith C., Andrews J., Reed K., Smith M., Millar C., Lambert D., Sinclair A. (2000). ASW: A gene with conserved avian W-linkage and female specific expression in chick embry- onic gonad. Dev. Genes Evol..

[25] Suh A., Kriegs J.O., Brosius J., Schmitz J. (2011). Retroposon insertions and the chronology of avian sex chromosome evolution. Mol. Biol. Evol..

[26] de Kloet R.S., de Kloet S.R. (2003). Evolution of the spindlin gene in birds: Independent cessation of the recombination of sex chromosomes at the spindlin locus in neognathous birds and tinamous, a palaeognathous avian family. Genetica.

[27] Li W., Xue F., Li L., Li X., Yue B., Li J. (2012). A triple-primer PCR approach for the sex identification of endangered Phasianidae birds. Eur. J. Wildl. Res..

[28] Kroczak A., Wołoszyńska M., Wierzbicki H., Kurkowski M., Grabowski K.A., Piasecki T., Urantówka A.D. (2021). New Bird sexing strategy developed in the order Psittaciformes involves multiple markers to avoid sex misidentification: Debunked myth of the Universal DNA marker. Genes.

[29] Kroczak A., Wierzbicki H., Urantówka A.D. (2022). In Silico Analysis of Seven PCR Markers Developed from the *CHD1*, *NIPBL* and *SPIN* Genes Followed by Laboratory Testing Shows How to Reliably Determine the Sex of Musophagiformes Species. Genes.

[30] Medeiros R.J., King R.A., Symondson W.O., Cadiou B., Zonfrillo B., Bolton M., Thomas R.J. (2012). Molecular evidence for gender differences in the migratory behaviour of a small seabird. PLoS ONE.

[31] Çakmak E., Akın Pekşen Ç., Bilgin C.C. (2017). Comparison of three different primer sets for sexing birds. J. Vet. Diagn. Investig..

[32] Casey A.E., Jones K.L., Sandercock B.K., Wisely S.M. (2009). Heteroduplex molecules cause sexing errors in a standard molecular protocol for avian sexing. Mol. Ecol. Resour..

[33] Dawson D.A., Darby S., Hunter F.M., Krupa A.P., Jones I.L., Burke T. (2001). A critique of avian CHD-based molecular sexing protocols illustrated by a Z-chromosome polymorphism detected in auklets. Mol. Ecol. Notes.

[34] Zhang P., Han J., Liu Q., Zhang J., Zhang X. (2013). Sex Identification of Four Penguin Species Using Locus-Specific PCR. Zoo Biol..

[35] Faux C.E., McInnes J.C., Jarman S.N. (2014). High-throughput real-time PCR and melt curve analysis for sexing Southern Ocean seabirds using fecal samples. Theriogenology.

[36] He P.J., Yu J.Q., Fang S.G. (2005). Sex identification of the black swan (*Cygnus atratus*) using the locus-specific PCR and implications for its reproduction. Reprod. Domest. Anim..

[37] Turcu M.C., Paștiu A.I., Bel L.V., Cocostîrc V., Lucaci F., Pusta D.L. (2022). DNA Sex Identification Using Different Biological Samples from Four Companion Bird Species. Bull. Univ. Agric. Sci. Vet. Med. Cluj-Napoca. Hortic..

[38] Turcu M.C., Paștiu A.I., Bel L.V., Pusta D.L. (2023). A comparison of feathers and oral swab samples as DNA sources for molecular sexing in companion birds. Animals.

[39] Turcu M.C., Paștiu A.I., Bel L.V., Pusta D.L. (2023). Minimally invasive sampling methods for molecular sexing of wild and companion birds. Animals.

[40] Mazzoleni S., Němec P., Albrecht T., Lymberakis P., Kratochvíl L., Rovatsos M. (2021). Long-term stability of sex chromosome gene content allows accurate qPCR-based molecular sexing across birds. Mol. Ecol. Resour..

[41] Handel C.M., Pajot L.M., Talbot S.L., Sage G.K. (2006). Use of buccal swabs for sampling DNA from nestling and adult birds. Wildl. Soc. Bull..

[42] Rovatsos M., Vukić J., Lymberakis P., Kratochvíl L. (2015). Evolutionary stability of sex chromosomes in snakes. Proc. R. Soc. B.

[43] Cankar K., Štebih D., Dreo T., Žel J., Gruden K. (2006). Critical points of DNA quantification by real-time PCR–effects of DNA extraction method and sample matrix on quantification of genetically modified organisms. BMC Biotechnol..

